# Impact of Treatment with GLP1 Receptor Agonists, Liraglutide 3.0 mg and Semaglutide 1.0 mg, While on a Waiting List for Bariatric Surgery

**DOI:** 10.3390/biomedicines11102785

**Published:** 2023-10-13

**Authors:** Miguel A. Rubio-Herrera, Sara Mera-Carreiro, Andrés Sánchez-Pernaute, Ana M. Ramos-Levi

**Affiliations:** 1 Departament of Endocrinology and Nutrition, Hospital Clínico San Carlos, Instituto de Investigación Sanitaria del Hospital Clínico San Carlos (IdISSC), 28040 Madrid, Spain; 2 Department of Medicine, Faculty of Medicine, Universidad Complutense, 28040 Madrid, Spain; 3 Department of Surgery, Hospital Clínico San Carlos (IdISSC), Faculty of Medicine, Department of Surgery, Universidad Complutense, 28040 Madrid, Spain; pernaute@yahoo.com; 4 Departament of Endocrinology and Nutrition, Hospital La Princesa, Instituto de Investigación Princesa, Universidad Autónoma de Madrid, 28049 Madrid, Spain; ana_ramoslevi@hotmail.com

**Keywords:** severe obesity, bariatric surgery, liraglutide, semaglutide, waiting list

## Abstract

Background: Weight loss before undergoing metabolic and bariatric surgery (MBS) has been suggested to reduce perioperative complications, although with controversial results. The objective of this study is to evaluate the impact of treatment with GLP1-R agonists (liraglutide 3.0 mg and semaglutide 1.0 mg) on preoperative weight loss and patients’ decisions regarding MBS while on a surgical waiting list. Materials and methods: One hundred and two patients on a waiting list for MBS started treatment with GLP1-RA for at least 6 months. Changes in weight at 26 and 52 weeks, the number of patients achieving >5% weight loss, and patients’ decisions regarding MBS were evaluated. Results: After 52 weeks, patients lost 16.9 ± 7.2% of weight with semaglutide 1.0 mg and 16.1 ± 5.8% of weight with liraglutide 3.0 mg. All patients lost ≥5% of initial weight, 84.7% lost ≥10%, 54.6% lost ≥15%, and 27.5% reached ≥20%. A total of 68.6% of participants were satisfied with the achieved weight loss and withdrew from the waiting list for MBS. A threshold of >15.1% weight loss had the greatest sensitivity and specificity for the final decision regarding undergoing MBS. Conclusions: Losing >15% of initial weight after 52 weeks of treatment with liraglutide 3.0 mg or semaglutide 1.0 mg during the waiting list for MBS impacts patients’ decisions regarding the final acceptance or rejection of the procedure.

## 1. Introduction

Metabolic and bariatric surgery (MBS) has been proven to be a safe and effective treatment for severe obesity (BMI > 35 kg/m^2^) and its associated comorbidities and all-cause mortality [1]. Moderate weight loss (5–10%) is enough to achieve a significant improvement in accompanying cardiovascular risk factors, but sustained weight loss is one of the greatest challenges in the management of obesity [2].

Numerous healthcare insurance plans call for a minimum of 5–15% weight loss before undergoing MBS to provide financial coverage in their attempt to limit the indications and reduce access to bariatric procedures. However, there are no randomized clinical trials, prospective studies, or meta-analyses that support preoperative weight loss as an essential prerequisite. In fact, the 1991 NIH Consensus Statement on the Treatment of Obesity [3] did not suggest the need for weight loss prior to undergoing MBS. In addition, the authors of the Updated Position Statement on Insurance Mandated Preoperative Weight Loss Requirements of the American Society for Metabolic and Bariatric Surgery (ASMBS) [4] considered this preoperative requirement arbitrary, discriminatory, and without scientific evidence, which only leads to delaying an effective surgical approach for the management of obesity and its life-threatening comorbid conditions. Indeed, the attrition rate may be high and entails more risks than benefits.

However, there have been studies that have attempted to evaluate the potential impact of acute preoperative weight loss on perioperative and postsurgical outcomes, including the reduction in liver volume, intraoperative bleeding, anastomotic leakage, deep infections, mean hospital stay or postsurgical complications, or even trying to evaluate its utility as a predictor of surgical success. In this regard, data derived from systematic reviews and meta-analyses conclude that preoperative weight loss may have a modest impact on perioperative issues, but there is no clear evidence supporting the effectiveness of several different weight loss intervention programs on long-term postoperative weight loss [5,6]. Moreover, highly experienced surgical teams that use advanced technology, such as laparoscopic and robotic-assisted approaches, have such low complication rates that the beneficial effect of preoperative weight loss to reduce postoperative complications becomes almost insignificant [7].

Pharmacological treatment for obesity has been mainly focused on the setting of insufficient or inadequate postsurgical weight loss, weight plateau, or post-surgical weight regain [8,9,10]. In this regard, GLP1 receptor agonists have been the In this regard, GLP1 receptor agonists have been one of the most frequently evaluated drugs for weight treatment, but data are limited to small retrospective and observational studies with short-term follow-up (less than 6 months), reaching 3.4–9.7% weight loss, depending on the dose used [11,12,13,14,15]. In the few studies that followed up patients for up to 12 months, mean weight loss reached 14–17% [16,17,18]. The only randomized clinical trial comparing liraglutide 3.0 mg versus a placebo, after weight regain, was carried out in 70 patients with poor weight response following gastric bypass or sleeve gastrectomy and a follow-up of 24 weeks. The results showed that liraglutide 3.0 mg was better than the placebo in achieving weight loss: −8.82 ± 4.94% vs. −0.54 ± 3.32%; *p* < 0.001 [19]. Overall, the results have proven to be similar to real-world studies in patients who have not undergone prior bariatric surgery [20,21,22].

Data regarding the use of anti-obesity drugs for the preoperative management of patients are rather limited. There are studies with orlistat 60 mg three times per day, sibutramine, topiramate–fluoxetine combinations, and extended-release phentermine–topiramate, attempting to achieve 10% weight loss prior to bariatric surgery [23,24,25], but to our knowledge, and to date, GLP1 receptor agonists have not been specifically evaluated for this preoperative indication. The European Medicines Agency (EMA) has authorized the commercialization of the GLP1 receptor agonist liraglutide 3.0 mg for patients with obesity, and semaglutide 0.25, 0.5, and 1 mg and dulaglutide 0.75 and 1.5 mg for patients with obesity and type 2 diabetes. Semaglutide 2.4 mg has been also approved by the EMA for the treatment of obesity, but its commercialization is still to come.

The objective of our study is to analyze the effect of liraglutide 3.0 mg and semaglutide 1.0 mg on preoperative weight loss in MBS candidates awaiting the procedure, as well as to evaluate the impact of pre-surgical weight loss on patients’ final decisions regarding acceptance or rejection to undergoing surgery.

## 2. Materials and Methods

### 2.1. Study Design

We performed a single-center retrospective observational study. The study was approved by the Ethics Committee of the Hospital Clínico San Carlos (code: 23/581-O_M_NoSP), and is in compliance with the Helsinki Declaration.

### 2.2. Subjects

During the years 2019–2022, 102 consecutive patients with severe obesity (BMI ≥ 40 kg/m^2^ or BMI ≥ 35 kg/m^2^ with associated comorbidities), aged 18–65 years, eligible for MBS, who were scheduled for the procedure with a waiting list of more than 12 months, were offered the possibility of initiating pharmacological treatment during their waiting time in an attempt to improve potential obesity-related comorbidities until the surgical procedure was performed. Specifically, patients with type 2 diabetes were prescribed semaglutide 1.0 mg weekly, according to product label and public healthcare system funding. For individuals with obesity and no type 2 diabetes, liraglutide 3.0 mg daily was offered according to the product label, but because this treatment has no public healthcare grant, only patients who could afford a minimum 6-month treatment were included. Patients underwent the same treatment and follow-up as obese patients who were not eligible for MBS, thus resembling an approximation to a real-world study.

### 2.3. Treatment and Follow-Up

Participants with liraglutide 3.0 mg were instructed to dose escalate, starting with 0.6 mg once daily and increasing by 0.6 mg weekly until 3.0 mg was reached at week 5 or 6, depending on gastrointestinal tolerance. Similarly, individuals on semaglutide 1.0 mg were instructed to begin with a 0.25 mg weekly dose, and progressively titrate it after four weeks until 1.0 mg per week was reached at week 12. If after 3 months of treatment with the maximum tolerated dose a minimum 5% weight loss was not reached, treatment was withdrawn, according to the product label and indications. We did not include in our study patients who, once GLP1-RA was started, required adding another hypoglycemic agent potentially affecting weight (such as SGLT2-I, pioglitazone, or insulin) after inclusion in the study in order to avoid bias regarding the evaluation of GLP1-RAs’ efficacy. At each visit, a healthcare professional recorded any possible adverse effects and verified the titration of the drugs.

In parallel, all participants received lifestyle counseling (from qualified health care professionals) every 4–6 weeks, in person or by telephone, to improve adherence. Participants were prescribed a reduced-calorie diet (−600 kcal/d deficit relative to estimated energy expenditure calculated at week 0) and increased physical activity (>150 min/wk, such as brisk walking and strength exercises). Both diet and activity were recorded daily in a diary and were reviewed during counseling visits.

After 12 months of pharmacological treatment, when the waiting time for bariatric surgery came to an end, patients were offered to choose one of three options: (1) continue pharmacological treatment and withdraw from the bariatric surgery waiting list; (2) continue pharmacological treatment and reconsider undergoing surgery later on; or (3) withdraw pharmacological treatment and undergo bariatric surgery.

### 2.4. Main Outcomes and Measures

Co-primary endpoints were a percentage change in body weight from baseline to week 52 and an achievement of weight loss of at least 5% of baseline weight at weeks 26 and 52. Body weight was measured using a weighing scale (SECA 684), with participants wearing light clothes and no shoes, and rounding to the nearest 0.1 kg. The percentage of body weight loss was calculated as 100 × [(body weight at baseline − body weight at week 26 or 52)/body weight at baseline].

Metabolic secondary outcomes included change from baseline in glycemic indices (fasting glucose, insulin, HOMA-IR, and hemoglobin A1c), lipids (total cholesterol, low-density lipoprotein cholesterol, high-density lipoprotein cholesterol, non-HDL cholesterol, and triglycerides), and hepatic function (alanine aminotransferase (ALT), aspartate aminotransferase (AST) and gamma-glutamyl transferase (GGT)).

### 2.5. Statistical Analysis

Continuous variables were summarized as mean ± standard deviation. Categorical variables were expressed as absolute frequencies (percentages). The Shapiro–Wilk test was used to check the normality of the variable’s distribution. Comparison between continuous variables was performed using an independent-sample *t*-test. For variables with a skewed distribution, the Mann–Whitney U-test was used for mean comparisons. The chi-squared test was used to analyze categorical data.

Repeated-measures ANOVA was conducted for each outcome using time (moment of assessment) as a within-subjects factor and group (semaglutide vs. liraglutide) as a between-subjects factor. For the moment of assessment, only baseline, 6-month, and 12-month evaluations were included due to the presence of missing values in the visits at 3 and 9 months. Mauchly’s test was used to determine whether the assumption of sphericity was met, and Leven’s test was used to assess the homogeneity of variance. When a violation of sphericity was observed, Greenhouse–Geisser-corrected *p*-values were reported.

The change in laboratory variables in the whole sample was compared using the Wilcoxon signed-rank test.

Receiver operating characteristic (ROC) curves were calculated to evaluate the capacity of detecting patients who rejected bariatric surgery after pharmacological treatment. Youden’s index (YI) was estimated to evaluate the best cutoff points.

A *p*-value < 0.05 was considered statistically significant. All statistical analyses were performed using IBM^®^ SPSS 26.0, JASP Team (2023, version 0.17.1 computer software) and Jamovi (version 2.4.).

## 3. Results

### 3.1. Characteristics of the Sample

One hundred and two patients were included. The mean age was 52.88 ± 10.38 years, and 71 (69.3%) patients were women. A total of 35 patients were treated with semaglutide 1.0 mg, and 67 were treated with liraglutide 3.0 mg. As expected, the frequency of T2D was higher in the semaglutide group than in patients taking liraglutide (100% vs. 0%, X^2^ = 86.07, *p* < 0.001).

The main clinical and demographic characteristics are depicted in Table 1 for the whole sample and each therapeutic group. There were no statistically significant differences in weight and BMI at baseline. However, the semaglutide group was older and showed a higher prevalence of arterial hypertension and dyslipidemia. Other comorbidities, such as obstructive sleep apnea and knee osteoarthritis, showed no significant differences.

### 3.2. Efficacy of Pharmacological Treatment

**Figure 1:**
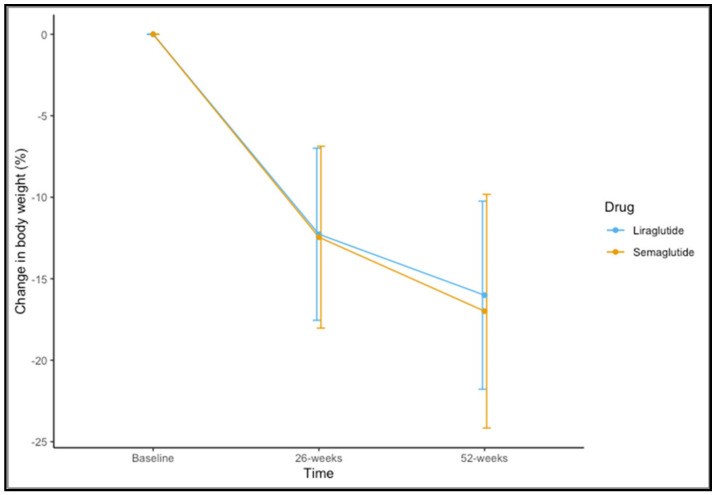
Changes in percentage of weight loss at 26 and 52 weeks for liraglutide and semaglutide.

Eighty-five (83.3%) participants completed 52 weeks of therapy. For the variable of weight, time-by-group interaction was not significant (F(1,83) = 0.437, *p* = 0.582). A significant main effect of time was observed (F(1,83) = 328.189, *p* < 0.001). The mean observed change in the percentage of weight loss is shown in Figure 1. The mean change in percentage weight loss at 52 weeks was 16.99 ± 7.17 for semaglutide 1.0 mg and 16.01 ± 5.77 for liraglutide 3.0 mg (*t* = 0.644, *p* = 0.522).

**Figure 2:**
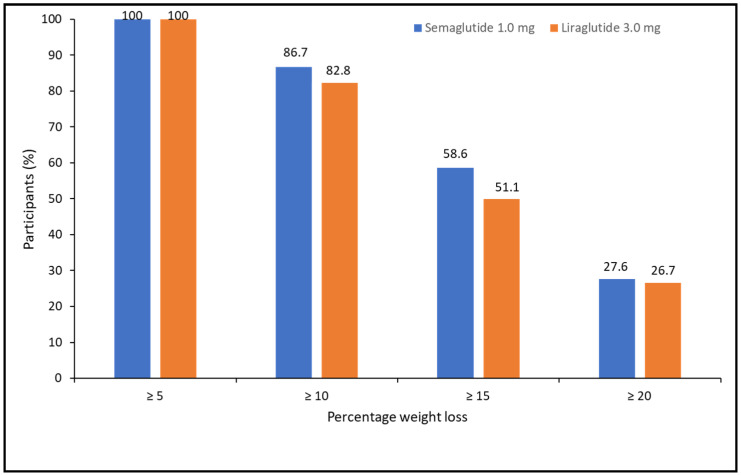
Categories of percentage of weight loss at 52 weeks of pharmacological treatment for semaglutide and liraglutide.

When categorizing the percentage of weight loss achieved at 52 weeks, 100% of patients lost ≥5%, 85.1% lost ≥10%, 54.1% lost ≥15%, and 27.5% lost ≥20%. There were no differences between treatments (X^2^ = 1.105, *p* = 0.576) (Figure 2).

**Figure 3:**
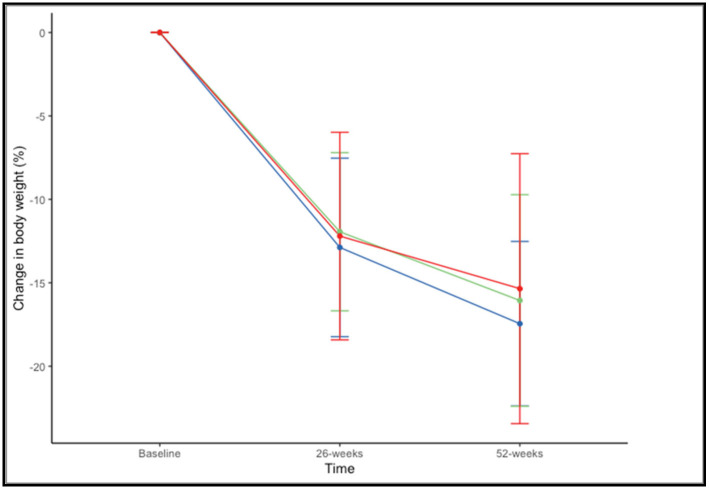
Changes in in percentage of weight loss at 26 and 52 weeks according to BMI range: 35–39.99 kg/m^2^ (blue), 40–44.99 kg/m^2^ (green), and >45 kg/m^2^ (red).

There were no significant differences in the percentage of weight loss at 52 weeks across BMI ranges (F(2,73) = 0.659, *p* = 0.520). The mean percentage of weight loss, considering both drugs together, was 17.45% ± 4.93% in patients with a BMI of 35–39.99 kg/m^2^, 16.06% ± 6.34% with a BMI of 40–44.99 kg/m^2^, and 15.35% ± 8.09% with BMI > 45 kg/m^2^ (Figure 3).

**Figure 4:**
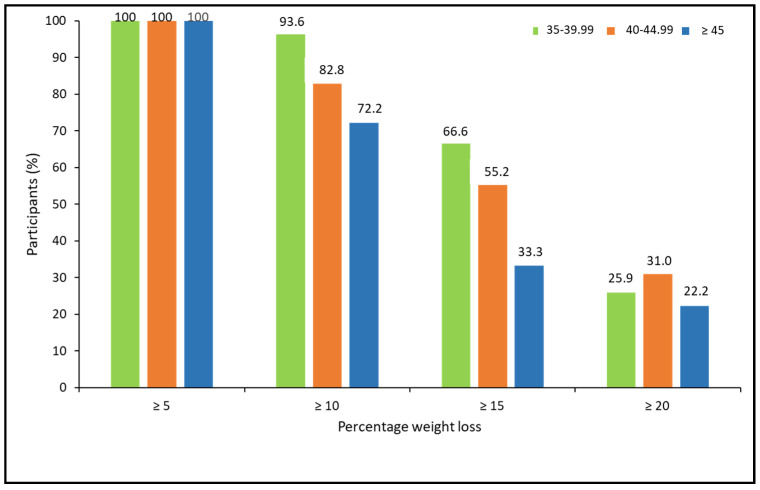
Categories of percentage of weight loss at 52 weeks post-pharmacological treatment according to BMI ranges.

When analyzing the percentage of weight loss achieved, according to the ranges of BMI, there were no statistically significant differences between BMI groups. However, a trend for a lower response rate for more ambitious weight loss goals was observed in patients with higher BMI (X^2^ = 5.15, *p* = 0.076 for ≥10%; X^2^ = 4.85, *p* = 0.088 for ≥15%; and X^2^ = 0.463, *p* = 0.793 for ≥20%; (Figure 4).

### 3.3. Adverse Events

Nausea was present in 33 (32.35%) and vomiting in 8 (7.43%), with no differences between semaglutide and liraglutide (28.57% vs. 34.32% X^2^ = 0.348, *p* = 0.555 for nausea, and 8.57% vs. 7.46%, X^2^ = 0.039, *p* = 0.843 for vomiting). Constipation was present in 16 (23.88%) cases under treatment with liraglutide, and no cases were reported with semaglutide (X^2^ = 9.91, *p* = 0.002). Conversely, diarrhea was more frequent in patients with semaglutide (6 (17.14%) vs. 1 (1.49%), X^2^ = 8.810, *p* = 0.003). Most adverse events were mild, limited in time, and did not imply treatment withdrawal.

### 3.4. Decision of Rejection of Bariatric Surgery

At week 52, 70 patients (68.62%) reported their intention to abandon the waiting list for bariatric surgery, while 32 (31.4%) were willing to undergo bariatric surgery. Among patients withdrawing from the waiting list, there were two levels of certainty in the decision: 52 (51%) expressed their intention to definitely abandon the plan for surgery, while 18 (17.6%) did not exclude this possibility in the future. There were no statistically significant differences in the final decision to reject surgery plans between patients taking liraglutide (20, 29.9%) or semaglutide (12, 34.3%) (X^2^ = 0.210, *p* = 0.647). The baseline and follow-up characteristics of groups withdrawing or not from bariatric surgery plans are shown in Table 2. These results are also shown according to the drug used (semaglutide 1.0 mg or liraglutide 3.0 mg) in Supplementary Table S1. Patients remaining on a bariatric surgery waiting list showed a greater BMI at baseline and worst response to pharmacological treatment at 6 and 12 months.

### 3.5. ROC Curve Analysis

**Figure 5:**
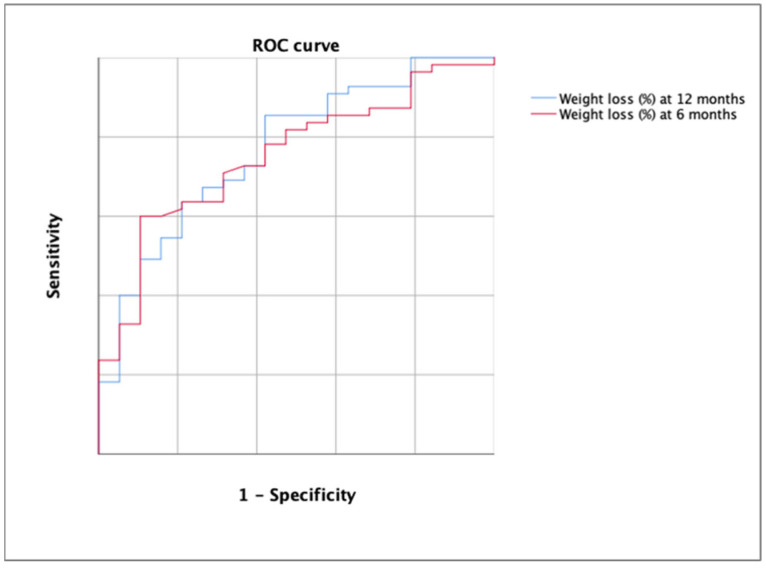
ROC curve for the detection of the rejection decision of bariatric surgery using the percentage of weight loss at 6 months (in red) and at 12 months (in blue). Each line represents 0.2 of sensitivity or 1-specificity.

The AUC for the detection of the rejection decision of bariatric surgery using the percentage of weight loss was 0.752 (*p* = 0.001) at 6 months and 0.768 at 12 months (Figure 5).

According to DeLong’s test, there were no significant differences between both ROC curves (*p* > 0.05). The best cutoff in the first case was 12.13 (YI = 0.384; sensitivity, 57.14; specificity, 81.25%) and 15.12 in the second case (YI = 0.426; sensitivity, 63.64%; specificity, 78.95%). All cutoff points are shown in Table 3.

### 3.6. Changes in Laboratory Parameters

During the study, we found statistically significant changes in the metabolic parameters associated with glycemic control, atherogenic dyslipidemia, insulin resistance, and ALT and GGT concentrations (Table 4). Supplementary Table S2 shows separate laboratory outcomes for semaglutide 1.0 mg and liraglutide 3.0 mg. Supplementary Table S2 shows laboratory outcomes for semaglutide 1.0 mg and liraglutide 3.0 mg. In the subgroup of 36 patients with T2D and the available laboratory data, the percentage of patients reaching HbA1C < 6.5% changed from 59% at baseline to 82.1% at the end of the study. When considering the goal of HbA1c < 5.7%, the percentage at baseline was 12.8% and 53.8% after 1 year of pharmacological treatment.

## 4. Discussion

Our results show that 68.6% of patients with obesity awaiting MBS who were treated with GLP1-receptor agonists reconsidered their decision and rejected undergoing the initial plan for a surgical approach. A percentage weight loss >15% after 52 weeks of treatment resulted in 63.64% sensitivity and 78.95% specificity for the final decision reached. Among patients withdrawing from the waiting list, 17.6% were uncertain and did not express if they would definitely remain on the MBS waiting list or if they would eventually withdraw. For the time being, they expressed their willingness to continue pharmacological treatment, but long-term economic issues, the status of their associated comorbidities, and their perceived quality of life will surely influence their final decision.

Metabolic and bariatric surgery is undoubtedly the most efficient long-term treatment for severe obesity, especially in terms of weight loss and the amelioration of associated comorbidities [1]. However, waiting lists may significantly hamper its accessibility in public healthcare systems. For instance, in Spain, in 2018, waiting time ranged between 397 and 1661 days [26]. Unfortunately, delaying bariatric procedures contributes to increasing obesity-related morbidity and mortality if no other approach is established [26,27,28,29].

The consensus statement of the American Society of Metabolic and Bariatric Surgery (ASMBS) and the International Federation for the Surgery of Obesity and Metabolic Disorders (IFSO) described the current indications for MBS [30]. Specifically, MBS is recommended for individuals with a body mass index (BMI) > 35 kg/m^2^, regardless of the presence, absence, or severity of comorbidities, and should be considered for individuals with metabolic disease and a BMI of 30–34.9 kg/m^2^. However, there is increasing clinical evidence of the usefulness of attempting a pharmacological approach prior to undergoing MBS [31]. For this purpose, in Spain, as in the rest of the European Union, GLP1-receptor agonists are the only commercialized drugs. We offered our patients liraglutide 3.0 mg for those with obesity but no diabetes and semaglutide 1.0 mg for those with both medical conditions. Additionally, in both cases, we encouraged the modification of lifestyle habits to try to maximize weight loss and ameliorate associated comorbidities and the overall quality of life. Unfortunately, semaglutide 2.4 mg is still under consideration for approval for commercialization by the EMA, and tirzepatide is not yet EMA-approved for obesity treatment.

The pivotal clinical trial with liraglutide 3.0 mg in patients with obesity and prediabetes showed a mean percentage weight loss of 8.0 ± 6.7% after 56 weeks [32] and reached 10.8% in early responders, defined as a weight loss >4% at 16 weeks [33]. Similarly, in the clinical trial with semaglutide 1.0 mg in patients with type 2 diabetes, the mean percentage of weight loss reached 6.5% [34]. In our study, the percentage of weight loss achieved with liraglutide 3.0 mg and semaglutide 1.0 mg was relatively similar, at both 6- and 12-month follow-up, reaching around 16% at the end of treatment, and clearly superior to the clinical trials mentioned above. Reasons for this difference may be related to the close follow-up performed in our patients and carrying out the lifestyle change advice, which is reinforced via the effects of appetite reduction and satiety induced by the GLP1-receptor agonists, as well as the motivation of patients awaiting MBS or because obese patients must bear the cost of their medication. Nevertheless, in real-world studies, similar weight loss rates have been reported after 6 and 12 months of treatment [16,17,18,35,36].

Current indications on the management of obesity suggest that at least 10% weight loss should be reached in order to significantly improve associated comorbidities. This therapeutic recommendation seems to include one of the two following definitions: (1) the ability to safely produce an average of >10% placebo-subtracted weight loss in randomized clinical trials in the majority of patients, or (2) the ability to safely produce a ≥15% weight loss in over half the patients as an adjunct to lifestyle [37]. In our study, more than 80% of participants reached a weight loss of more than 10%, more than half of the patients achieved a weight loss greater than 15%, and one out of four participants lost more than 20% of the initial weight. Indeed, these data are similar to what has been reported in prior randomized clinical studies with more potent drugs, such as semaglutide 2.4 mg in obese patients [38] or tirzepatide in patients with obesity and type 2 diabetes [39].

Interestingly, 68.6% of patients, who had lost around 15% of their initial body weight, decided to withdraw from the waiting list for MBS without any influence from the physician. In this regard, the threshold that best determined the highest sensitivity and specificity for the final decision to either continue with the surgical plan or drop out was a weight loss of 15.12% (63.64% sensitivity and 78.95% specificity, AUC ROC of 0.768), although this was observed after 6 months when weight loss was above 12%. To our knowledge, this is the first time that a sustained weight loss over a period of at least 26 weeks has had such a remarkable influence on the decision regarding the planification of MBS.

GLP1-receptor agonists entail significant metabolic changes beyond mere weight loss, particularly regarding the improvement in insulin sensitivity and lipid and hepatic profiles. The positive changes were also evident for individuals with type 2 diabetes, in whom the percentage of patients reaching different HbA1c goals improved.

Finally, 31.4% of participants remained keen to undergo a bariatric procedure. A higher baseline BMI and a poorer response to medical treatment contributed to persisting in the decision to undergo MBS. According to the logistic regression analysis performed in our study, age, sex, or comorbidities did not influence patients’ decisions. A percentage weight loss lower than 10% and, although to a lesser extent, a higher baseline BMI were the two main factors that contributed to the decision to undergo MBS. Weight loss could not be considered insufficient in patients who maintained their decision to undergo surgery, as it was truly in the range of prior clinical trials [32,33,34], and even though this efficacy lies around half the efficacy of MBS, it seemed insufficient for these patients so as to overcome the surgical decision.

In this setting, new drugs for the management of obesity, which are currently being evaluated in phase 3 studies, and that have proven to achieve weight loss rates above 20%, will almost reach comparable results to MBS [40,41,42] and will probably entail significant reductions in the long waiting lists for MBS. In any case, the main challenges will still concern patients’ abilities to sustain the cost of pharmacological long-term treatments, the economic restrictions of public healthcare systems and private healthcare insurance companies, and the ability to guarantee a regular market supply of such drugs if administration remains as weekly injections. In this regard, it is possible that oral formulations as tablets, with optimal efficacy results and comparable adverse effects to injectable formulations [43], could favor storage, stocking, distribution, supply, and cost reduction and, as a result, allow better accessibility to the millions of people living with obesity that will eventually need pharmacological treatment.

The secondary adverse effects reported by patients in our study were similar to the ones reported in the majority of prior clinical studies. Specifically, the main complaints concerned mild- to moderate-intensity gastrointestinal issues, which were mainly transitory and did not determine the need to withdraw medication.

Our study has some limitations. First, it is a single-center observational retrospective study, so the results cannot be fully transposed worldwide to what may happen in other hospitals. Second, only patients who could afford the cost of sustained pharmacological treatment were included, leaving out other patients with worse socio-economic situations. It is true that in the United Kingdom, financial support for the treatment with semaglutide 2.4 mg for 2 years may be available in patients with severe obesity [44], but this measure has not implied any new actions by healthcare authorities in the rest of Europe.

However, we remark on the strengths of our study. Specifically, it reflects what may occur in real-world clinical practice in patients who are keen to lose weight before undergoing MBS. These patients were closely followed up in a specific outpatient obesity clinic, with the support of trained nurses and registered dietitians, which warrants high adherence to long-term follow-up and medical treatment.

## 5. Conclusions

This study shows that a weight loss greater than 15% after one year of pharmacological treatment with GLP-1 receptor agonists may determine a shift in the decision to undergo MBS in around two-thirds of patients on a waiting list for a bariatric procedure. Promoting pharmacological treatment before MBS may help patients decide if they truly want to undergo a bariatric procedure.

## Figures and Tables

**Table 1:** Demographic characteristics, comorbidities, and laboratory tests at baseline and according to the type of pharmacological treatment received (semaglutide 1.0 mg or liraglutide 3.0 mg).

Characteristics	Semaglutide 1.0 mg (n = 35)	Liraglutide 3.0 mg (n = 67)	Statistic (*p*-Value)
Age, years	57.22 ± 5.79	50.61 ± 11.50	3.19 (0.002) ^a^
Sex, female (%)	60.0	74.62	2.32 (0.127) ^b^
Body weight, kg	117.77 ± 13.80	119.60 ± 29.47	−0.34 (0.729) ^a^
BMI, kg/m^2^	43.05 ± 4.25	43.92 ± 8.14	−0.58 (0.557) ^a^
BMI 35–39.99 n (%)	10 (28.6)	24 (35.8)	0.661 (0.719) ^b^
BMI 40–44.99 n (%)	15 (42.9)	24 (35.8)	
BMI ≥ 45 n (%)	10 (28.6)	19 (28.4)	
Comorbidities			
Arterial hypertension (%)	77.1	38.80	13.53 (<0.001) ^b^
Dyslipidemia (%)	54.28	31.3	5.07 (0.024) ^b^
Obstructive sleep apnea (%)	28.57	17.91	1.54 (0.214) ^b^
Knee osteoarthritis (%)	22.85	22.38	0.003 (0.957) ^b^
Laboratory tests *			
Glycemia (mg/dL)	133.96 ± 43.40	100.70 ± 13.89	1221 (<0.001) ^c^
HbA1c (%)	6.82 ± 1.48	5.61 ± 0.54	1194 (<0.001) ^c^
Insulin (µUI/mL)	26.07 ± 12.76	20.27 ± 14.95	578 (0.031) ^c^
HOMA-IR	8.26 ± 4.86	5.22 ± 4.10	613 (0.007) ^c^
Total cholesterol, mg/dL	167.53 ± 47.36	191.55 ± 43.05	572 (0.074) ^c^
Non-HDL cholesterol	119.68 ± 44.73	141.46 ± 40.55	587 (0.099)^c^
HDL-c, mg/dL	47.84 ± 8.99	50.08 ± 12.67	639 (0.261) ^c^
LDL-c, mg/dL	92.12 ± 36.89	114.17 ± 34.94	499 (0.020) ^c^
Triglycerides, mg/dL	155.96 ± 99.69	134.40 ± 59.83	809 (0.573) ^c^
AST, U/L	24.25 ± 9.63	24.27 ± 13.10	797 (0.653) ^c^
ALT, U/L	26.81 ± 15.22	26.02 ± 19.76	829 (0.441) ^c^
GGT, U/L	37.87 ± 22.51	34.74 ± 34.83	943 (0.057) ^c^

^a^: Student’s *t*; ^b^: X^2^-squared; ^c^: Mann–Whitney’s U. * Laboratory tests were available in a subgroup of 77 patients.

**Table 2:** Baseline and 12-month follow-up characteristics of patients according to the decision of rejection of bariatric surgery after 52 weeks of pharmacological therapy.

Characteristics	Abandoning Bariatric Surgery Plans (n = 70)	Bariatric Surgery (n = 32)	Statistic (*p*-Value)
Age, years	53.94 ± 10.18	50.56 ± 10.58	1.536 (0.128) ^a^
Sex, female (%)	53 (75.7%)	18 (56.3%)	3.933 (0.047) ^b^
Baseline body weight, kg	113.94 ± 17.78	129.98 ± 34.27	−2.498 (0.017) ^a^
Baseline BMI, kg/m^2^	42.50 ± 5.22	46.07 ± 9.59	−1.970 (0.056) ^a^
52-week BMI, kg/m^2^	34.75 ± 4.88	39.95 ± 8.20	−3.597 (0.001) ^a^
BMI 35–39.99 n (%)	27 (38.6)	7 (21.9)	4.282 (0.118) ^b^
BMI 40–44.99 n (%)	27 (38.6)	12 (37.5)	
BMI ≥ 45 n (%)	16 (22.9)	13 (40.6)	
Comorbidities			
Type 2 DM (%)	37.1	40.6	0.113 (0.737) ^b^
Hypertension (%)	48.6	59.4	1.027 (0.311) ^b^
Dyslipidemia (%)	38.7	40.6	0.039 (0.844) ^b^
Obstructive sleep apnea (%)	21.4	21.9	0.003 (0.959) ^b^
Knee osteoarthritis (%)	21.4	25.0	0.160 (0.689) ^b^
WL (%)			
26 weeks	−13.51 ± 5.61	−9.75 ± 3.65	626 (<0.001)
52 weeks	−17.81 ± 6.32	−12.31 ± 4.37	242 (0.001)
≥5%	100	100	-
≥10%	92.7	63.2	9.757(0.002) ^b^
≥15%	63.6	26.3	7.920 (0.005) ^b^
≥20%	34.5	5.3	6.140 (0.013) ^b^
Laboratory tests (at baseline)			
Glycemia (mg/dL)	109.28 ± 21.16	124.15 ± 49.47	595 (0.334) ^c^
HbA1c (%)	5.98 ± 1.08	6.40 ± 1.39	559 (0.385) ^c^
Insulin (µUI/mL)	22.99 ± 15.03	23.47 ± 11.69	315 (0.627) ^c^
HOMA-IR	6.36 ± 4.59	7.67 ± 5.03	285 (0.318) ^c^
Total cholesterol, mg/dL	182.32 ± 44.74	180.80 ± 49.65	671 (0.855) ^c^
Non-HDL cholesterol	132.31 ± 42.22	133.30 ± 46.47	688 (0.992) ^c^
HDL-c, mg/dL	50.00 ± 12.50	47.50 ± 8.37	585 (0.280) ^c^
LDL-c, mg/dL	104.67 ± 34.76	106.96 ± 42.38	650 (0.898) ^c^
Triglycerides, mg/dL	135.86 ± 61.04	157.96 ± 105.70	635 (0.577) ^c^
AST, U/L	26.11 ± 13.49	20.50 ± 5.47	510 (0.061) ^c^
ALT, U/L	27.49 ± 21.18	24.00 ± 8.04	636 (0.580) ^c^
GGT, U/L	36.62 ± 34.80	34.76 ± 18.63	610 (0.413) ^c^
Laboratory tests (at 12 months)			
Glycemia (mg/dL)	99.96 ± 14.25	107.52 ± 24.79	517 (0.149)
HbA1c (%)	5.49 ± 0.41	5.76 ± 0.82	530 (0.290)
Insulin (µUI/mL)	14.73 ± 9.40	13.70 ± 7.26	327 (0.876)
HOMA-IR	3.71 ± 2.58	5.95 ± 8.85	296 (0.486)
Total cholesterol, mg/dL	172.38 ± 36.93	178.17 ± 45.62	574 (0.576)
Non-HDL cholesterol	120.09 ± 34.31	125.43 ± 42.03	553 (0.609)
HDL-c, mg/dL	52.29 ± 11.85	49.39 ± 9.13	498 (0.250)
LDL-c, mg/dL	98.06 ± 31.03	105.28 ± 35.62	495 (0.362)
Triglycerides, mg/dL	114.65 ± 61.44	124.65 ± 75.94	577 (0.809)
AST, U/L	22.35 ± 8.96	21.24 ± 7.21	623 (0.872)
ALT, U/L	22.27 ± 10.24	22.16 ± 8.32	614 (0.799)
GGT, U/L	28.63 ± 24.23	28.42 ± 17.67	591 (9.811)

^a^: Student’s *t*; ^b^: X^2^-squared; ^c^: Mann–Whitney’s U.

**Table 3:** Sensitivity and specificity at different cutoff points at 26 and 52 weeks.

Percentage of Weight Loss at 26 Weeks							
Cutoff Point	Sensitivity (%)	Specificity (%)	PPV (%)	NPV (%)	Youden’s Index	AUC	Metric Score
10.17	72.86%	62.5%	80.95%	51.28%	0.354	0.720	0.354
10.29	71.43%	65.62%	81.97%	51.22%	0.371	0.720	0.371
10.3	70%	65.62%	81.67%	50%	0.356	0.720	0.356
10.6	68.57%	65.62%	81.36%	48.84%	0.342	0.720	0.342
11.84	60%	75%	84%	46.15%	0.350	0.720	0.350
12.06	58.57%	75%	83.67%	45.28%	0.336	0.720	0.336
12.08	57.14%	78.12%	85.11%	45.45%	0.353	0.720	0.353
12.13	57.14%	81.25%	86.96%	46.43%	0.384	0.720	0.384
12.2	55.71%	81.25%	86.67%	45.61%	0.370	0.720	0.370
12.23	54.29%	81.25%	86.36%	44.83%	0.355	0.720	0.355
12.42	52.86%	81.25%	86.05%	44.07%	0.341	0.720	0.341
Percentage of weight loss at 52 weeks							
Cutoff Point	Sensitivity (%)	Specificity (%)	PPV (%)	NPV (%)	Youden’s Index	AUC	Metric Score
11.48	85.45%	57.89%	85.45%	57.89%	0.433	0.768	0.433
11.53	83.64%	57.89%	85.19%	55%	0.415	0.768	0.415
11.78	81.82%	57.89%	84.91%	52.38%	0.397	0.768	0.397
14.39	67.27%	73.68%	88.1%	43.75%	0.410	0.768	0.410
14.66	65.45%	73.68%	87.8%	42.42%	0.391	0.768	0.391
15.12	63.64%	78.95%	89.74%	42.86%	0.426	0.768	0.426
15.45	61.82%	78.95%	89.47%	41.67%	0.408	0.768	0.408
15.88	60%	78.95%	89.19%	40.54%	0.389	0.768	0.389
16.67	54.55%	84.21%	90.91%	39.02%	0.388	0.768	0.388
17.46	49.09%	89.47%	93.1%	37.78%	0.386	0.768	0.386

**Table 4:** Baseline and follow-up laboratory parameters in the whole sample *.

Laboratory Tests	Baseline	At 12 Months	Z (*p*-Value)
Glycemia (mg/dL)	114.18 ± 33.65	102.42 ± 18.52	−4.13 (<0.001)
HbA1c (%)	6.12 ± 1.19	5.58 ± 0.58	−5.01 (<0.001)
Insulin (µUI/mL)	23.12 ± 14.10	14.44 ± 8.81	−5.09 <0.001)
HOMA-IR	6.71 ± 4.71	4.33 ± 5.14	
Total cholesterol, mg/dL	181.82 ± 46.10	174.21 ± 39.66	−2.52 (0.012)
Non-HDL cholesterol	132.64 ± 43.37	121.73 ± 36.64	−3.39 (0.001)
HDL-c, mg/dL	49.17 ± 11.32	51.40 ± 11.11	−1.48 (0.137)
LDL-c, mg/dL	105.41 ± 37.11	100.21 ± 32.38	−1.36 (0.174)
Triglycerides, mg/dL	143.13 ± 78.57	117.72 ± 65.86	−3.59 (<0.001)
AST, U/L	24.27 ± 11.75	21.99 ± 8.39	−1.74 (0.081)
ALT, U/L	26.34 ± 17.96	22.24 ± 9.59	−2.57 (0.010)
GGT, U/L	36.01 ± 30.32	28.56 ± 22.22	−4.02 (<0.001)

* Data available for 77 patients.

## Data Availability

Not applicable.

## Supplementary Materials

The following supporting information can be downloaded at: https://www.mdpi.com/article/10.3390/biomedicines11102785/s1, Table S1: Characteristics of patients according to the decision of abandoning bariatric surgery after 52 weeks of pharmacological therapy with semaglutide 1.0 mg and liraglutide 3.0 mg. Table S2: Baseline and follow-up laboratory parameters* according to the type of pharmacological treatment received.
