# Supplementary material for: Impact of Treatment with GLP1 Receptor Agonists, Liraglutide 3.0 mg and Semaglutide 1.0 mg, While on a Waiting List for Bariatric Surgery
Source: Biomedicines. 2023 Oct 13;11(10):2785. doi: 10.3390/biomedicines11102785 (PMC10604375; doi:10.3390/biomedicines11102785)
Supplement: Supplementary file 1 [file biomedicines-11-02785-s001.zip › biomedicines-2639354-supplementary.pdf]

SUPPLEMENTARY APPENDIX

Rubio-Herrera MA, Mera-Carreiro S, Sánchez-Pernaute A, Ramos-Leví AM. Impact of treatment with GLP1 receptor agonists, liraglutide 3.0 mg and semaglutide 1.0 mg, while on a waiting list for bariatric surgery. Biomedicines 2023

Table S1. Characteristics of patients according to the decision of abandoning bariatric surgery after 52 weeks of pharmacological therapy with semaglutide 1.0 mg and liraglutide 3.0 mg

**Characteristics**	**Semaglutide 1.0 mg (n=35)**		**Liraglutide 3.0 mg (n=67)**	
	**Abandoning bariatric surgery plans (n=23)**	**Bariatric Surgery (n=12)**	**Abandoning bariatric surgery plans (n=47)**	**Bariatric Surgery (n=20)**
Age, years	57.75±6.53	56.25±4.11	52.09±11.16	47.15±11.85
Sex, female (%)	15 (65.2%)	6 (50%)	38 (80.9%)	12 (60.0%)
Baseline Body weight, kg	117.23±13.81	118.80±14.35	112.33±19.36	136.69±40.88
Baseline BMI, kg/m2	43.07±4.16	43.01±4.62	42.23±5.69	47.90±11.32
52wk-BMI, kg/m^2	35.21±5.07	36.55±6.10	34.53±4.83	41.98±8.79
		Comorbidities		
Type 2 DM (%)	23 (100%)	12 (100%)	3 (6.4%)	1 (5.0%)
Hypertension (%)	18 (78.3%)	9 (75.0%)	16 (34.0%)	10 (50%)
Dyslipidemia (%)	11 (47.8%)	8 (66.7%)	16 (34.0%)	5 (25.0%)
Obstructive Sleep Apnea (%)	7 (30.4%)	3 (25.0%)	8 (17.0%)	4 (20.0%)
Knee Osteoarthritis (%)	4 (17.4%)	4 (33.3%)	11 (23.4%)	4 (20.0%)
		WL(%)		
26-wk	-13.43±6.30	-10.57±3.31	-13.55±5.32	-9.25±3.84
52-wk	-18.33±7.73	-14.01±4.86	-17.51±5.45	-10.78±3.42

Table S2. Baseline and follow-up laboratory parameters* according to the type of pharmacological treatment received.

	**Liraglutide (n=45)**		**Semaglutide (n=32)**	
**Laboratory tests**	*Baseline*	*At 12 months*	*Baseline*	*At 12 months*
Glycaemia (mg/dL)	100.70 ± 13.89	95.67 ± 9.01	133.97 ± 43.40	111.91 ± 23.80
HbA1c (%)	5.61 ± 0.54	5.50 ± 0.59	6.82 ± 1.48	5.68 ± 0.55
Insulin (µUI/mL)	20.27 ± 14.95	13.86 ± 9.66	26.07 ± 12.76	15.02 ± 8.00
HOMA-IR	5.22 ± 4.09	4.49 ± 6.90	8.26 ± 4.86	4.16 ± 2.46
Total cholesterol, mg/dL	191.55 ± 43.05	180.75 ± 39.83	167.53 ± 47.36	165.22 ± 38.22
Non-HDL cholesterol	141.46 ± 40.55	126.67 ± 36.86	119.68 ± 44.73	115.09 ± 35.85
HDL-c, mg/dL	50.08 ± 12.67	52.35 ± 11.06	47.84 ± 8.99	50.13 ± 11.22
LDL-c, mg/dL	114.17 ± 34.93	104.93 ± 32.15	92.12 ± 36.89	93.65 ± 32.06
Triglycerides, mg/dL	134.40 ± 59.83	108.70 ± 57.00	155.96 ± 99.69	129.84 ± 75.43
AST, U/L	24.28 ± 13.10	21.59 ± 8.36	24.25 ± 9.63	22.53 ± 8.54
ALT, U/L	26.02 ± 19.76	21.50 ± 7.87	26.81 ± 15.22	23.25 ± 11.61
GGT, U/L	34.74 ± 34.83	28.63 ± 26.79	37.87 ± 22.51	28.47 ± 14.36

*Data available for 77 patients.
